# 

**Published:** 1893-11-25

**Authors:** 


CONTENTS.

**The "Quarterly Review" on Hospitals** - 113
**Around the Hospitals** - 114
**On Modern Progress in Ophthalmic Medicine and Surgery.** By Robert Brudenell Carter, F.R.C.S. - 115
**The Medical Work of the Local Government Board.** V.—Diphtheria and Infective Endocarditis - 116
**Modern Aspects of Apendicitis.**—I. - 117
**The Hospital Clinic**—Some Remarks on Hernia. By Wheelton Hind, M.D., B.S., F.R.C.S. - 121
The Radical Cure of Hernia - 123
Royal Infirmary, Edinburgh—Treatment of Dilated Stomach 123
New Drugs and Preparations—Uric Acid Solvents in Gout and Gravel (*concluded*) - 124
New Appliances and Things Medical—Scotch Oatmeal - 124
**The Field of Science** - 116
**Annotations.**—Curiosities of Alcoholism—Kirkheaton Sanitary Matters—Death Certification and Murder - 119
**Notes and News** - 120
**The Institutional Workshop** — Hospital Construction—Nurses' Home, Edinburgh Royal Infirmary - 125
The Story of the Insane from Year to Year - 126
The Uniform System of Accounts for Hospitals and Institutions - 126
Hospital Meetings—British Home for Incurables, Clapham—Anniversary Meeting of the Salop Infirmary - 127
Construction Notes - 128
**Scraps and Gleanings** - 128
**The Book World of Medicine and Science** - vii

EXTRA SUPPLEMENT.—THE NURSING MIRROR.

**News from the Nursing World.**—Christmas Festivities—The Royal National Pension Fund—Misplaced Confidence—Our Christmas Competitions—Misplaced Parsimony—Drugs *versus* Common Sense—Conflicting Interests—Free of Debt—Inadequate Reforms—The Poor Ratepayers—Another Nurse Needed—Short Items - lxxi
**On the Nursing of Diseases of the Stomach.** III.—Vomiting (*continued*) - lxxiii
**Minor Appointments—Wants and Workers** - lxxiii
**Women's Work.**—Female Medical Aid for the Women of India. By Mrs. Scharlieb, M.D. II.—Medical Aid - lxxiv
**Notes and Queries** - lxxiv
**Nursing in the United States.**—Proper Organisation of Training Schools (*continued*). By Miss Darche - lxxv
**Appointments** - lxxv
**Everybody's Opinion.**—Asylum v. Hospital Training—A Searching Investigation Imperative - lxxvi
**Our Examination** - lxxvii
**Causerie from New England—Where to Go** - lxxvii
**The Muses' Looking Glass.**—"Utopia" at the Savoy Theatre lxxviii
**Novelty for Nurses.**—Irish Linens - lxxviii
**The Book World for Women and Nurses** - lxxix

